# O1.6. INCREASED COMPLEMENT FACTORS C3 AND C4 IN SCHIZOPHRENIA AND THE EARLY STAGES OF PSYCHOSIS: IMPLICATIONS FOR CLINICAL SYMPTOMATOLOGY AND CORTICAL THICKNESS

**DOI:** 10.1093/schbul/sby015.188

**Published:** 2018-04-01

**Authors:** Vanessa Cropley, Liliana Laskaris, Andrew Zalesky, Cynthia Shannon Weickert, Maria Di Biase, Gursharan Chana, Bernhard Baune, Chad Bousman, Barnaby Nelson, Patrick D McGorry, Ian Everall, Christos Pantelis

**Affiliations:** 1 Melbourne Neuropsychiatry Centre, The University of Melbourne; 2 Neuroscience Research Australia; 3 Centre for Neural Engineering, The University of Melbourne; 4 University of Adelaide; 5 University of Calgary; 6 Orygen, The National Centre of Excellence in Youth Mental Health; 7 Institute of Psychiatry, Psychology & Neuroscience, King’s College London

## Abstract

**Background:**

The complement system - a key component of the innate immune system, has been proposed to contribute to the pathogenesis of schizophrenia. Recently, complement C4 was associated with increased risk of schizophrenia, and in a mouse model, developmentally-timed synaptic pruning. These observations have led to proposals that abnormal activation of the complement system might contribute to the development of schizophrenia by disrupting synaptic pruning during key developmental periods. However, despite renewed interest in the complement system in schizophrenia it remains unclear whether peripheral complement levels differ in cases compared to controls, change over the course of illness and whether they are associated with current symptomatology and brain cortical thickness. This study aimed to: i) investigate whether peripheral complement protein levels are altered at different stages of illness, and ii) identify patterns among complement protein levels that predict clinical symptoms and grey matter thickness across the cortex.

**Methods:**

Complement factors C1q, C3 and C4 were quantified in 183 participants [n=83 Healthy Controls (HC), n=10 Ultra-High Risk (UHR) for psychosis, n=40 First Episode Psychosis (FEP), n=50 Chronic schizophrenia] using Multiplex ELISA. Permutation-based t-tests were used to assess between-group differences in complement protein levels at each of the three illness stages, relative to age- and gender-matched healthy controls. Canonical correlation analysis was used to identify patterns of complement protein levels that correlated with clinical symptoms and regional thickness across the cortex.

**Results:**

C3 and C4 were significantly increased in FEP and UHR patients, whereas only C4 was significantly increased in chronic patients. A molecular pattern of increased C4 and decreased C3 was associated with positive and negative symptom severity in the pooled patient sample. Increased C4 levels alone, or decreased C3 levels alone, did not correlate with symptom severity as strongly as the pattern of increased C4 in combination with decreased C3. Preliminary canonical correlation analyses revealed that, in healthy controls, a molecular pattern characterised by increased C3 and decreased C4 was associated with relatively thinner paracentral, inferior parietal and inferior temporal cortices, but relatively thicker insular, in the left hemisphere. In the pooled patient group, a trend for increased C3 in combination with decreased C1q was associated with relatively thinner left lateral occipital cortex and pars orbitalis but relatively thicker pars opercularis and precuneus.

**Discussion:**

Our findings indicate that peripheral complement concentration is particularly increased early and preceding psychosis and its imbalance may be associated with symptom severity and variation in regional grey matter thickness across the cortex.

